# Heterologous Challenge with PRRSV-1 MLV in Pregnant Vaccinated Gilts: Potential Risk on Health and Immunity of Piglets

**DOI:** 10.3390/ani12040450

**Published:** 2022-02-12

**Authors:** Georgios Papakonstantinou, Eleftherios Meletis, Georgios Christodoulopoulos, Eleni D. Tzika, Polychronis Kostoulas, Vasileios G. Papatsiros

**Affiliations:** 1 Clinic of Medicine, Faculty of Veterinary Medicine, School of Health Sciences, University of Thessaly, 43100 Karditsa, Greece; gc@vet.uth.gr (G.C.); vpapatsiros@vet.uth.gr (V.G.P.); 2 Laboratory of Epidemiology & Artificial Intelligence, Faculty of Public Health, School of Health Sciences, University of Thessaly, 43100 Karditsa, Greece; emeletis@outlook.com (E.M.); pkost@uth.gr (P.K.); 3 Farm Animals Clinic, School of Veterinary Medicine, Aristotle University of Thessaloniki, 54627 Thessaloniki, Greece; eltzika@vet.auth.gr

**Keywords:** PRRSV, antibodies, MLV vaccine, safety, piglet

## Abstract

**Simple Summary:**

Modified live virus (MLV) vaccines are considered as the key component to control the Porcine Reproductive and Respiratory Syndrome Virus (PRRSV). The majority of pig farms apply the ‘mass’ vaccination strategy in breeding female animals. However, this PRRS MLV vaccination protocol involves the risk of inoculation of sows in the last stage of gestation, resulting in possible infection of the fetus as the virus can efficiently cross the placenta during the last period of pregnancy. Thus, we evaluated the ability of the vaccine virus to act as a pathogenic strain, to be transmitted to fetuses and to affect the health status of neonatal piglets. The results indicated that the study gilts transmitted the vaccine virus to their offspring, as well as that the PRRSV-infected piglets showed a poor clinical performance. Consequently, the pig farms that apply PRRS MLV vaccination in a routine blanket vaccination strategy must avoid inoculating pregnant gilts the last week before their parturition.

**Abstract:**

The objective of the present study was to evaluate the potential risks of the four commercial PRRS-1 MLV vaccines in pregnant vaccinated gilts at the last stage of gestation under field conditions. The study was conducted at four pig farms, including 25 gilts from each farm (25 × 4 = 100 gilts), which were equally allocated to five different study groups. A PRRS-1 MLV vaccination was applied on the 100th day of their pregnancy with the different commercial vaccines that are available in the Greek market. The results indicated virus congenital infection and viremia in piglets (20/200 = 10% PRRSV infected piglets), and detection of PRRSV-specific antibodies (181/200 = 90.5% piglets found with PRRSV antibodies). The subsequent phylogenetic analyses revealed high percentages of similarity between the PRRSV-1 strain detected in infected litters and the PRRSV-1 vaccine strain to which the study gilts had been previously exposed to. Health status analyses of trial piglets resulted in differences between litters from vaccinated sows and litters from non-vaccinated sows at 110th day of gestation as regards the number of weak-born piglets, mummies, and piglets with splay-leg and/or respiratory symptoms. The current study’s results indicate several potential dangers of the PRRS MLV vaccination in late gestation.

## 1. Introduction

The porcine reproductive and respiratory syndrome virus (PRRSV) is considered to be one of the most challenging pig diseases having a significant economic impact on the swine industry. Since its first description in the early 1990s in Europe and North America, the virus has become endemic in most pig-breeding countries [1]. PRRS is a small enveloped, positive-sense, single-stranded RNA virus belonging to the order Nidovirales, family Arteriviridae [2,3,4]. Based on genetic, antigenic, and pathogenic differences, the virus can be classified into two genotypes: type 1, represented by the European subtypes (prototype Lelystad virus); and type 2, represented by the American subtypes [4,5,6]. Regarding the clinical manifestations of the viral infection in pigs, PRRS is responsible for reproductive failure in breeding animals (delayed estrus returns, abortions, and mummified or weak-born piglets) and respiratory disease in nursery, weaning, and growing—finishing pigs characterized by reduced growth performance and increased mortality rate, mostly in neonatal pigs [7,8,9,10]. The virus is also considered to be one of the most important pathogens causing the porcine respiratory disease complex (PRDC), which is associated with huge economic losses in the global pig industry [11]. Recent estimates from Europe and North America show that the reproductive performance of infected herds is decreased by approximately 1.4 weaner pigs/sow. To these figures the costs arising from the effects of an endemic infection on mortality and morbidity, daily weight gain, feed efficiency, and treatment costs should be added [12].

The high impact of PRRSV on the swine industry has caused the demand for the development of vaccines to control the disease in both growing and breeding animals. The PRRSV vaccines are based on modified live virus (MLV) or killed virus (KV) and they are currently commercially available for use in breeding pigs in several countries worldwide. Unfortunately, none of these were completely effective in preventing virus spreading within a herd [13,14,15]. KV vaccines do not induce virus-neutralizing antibodies and cannot prevent PRRS infections, even after challenge with homologous strains [16,17,18]. In contrast, MLVs are attenuated live vaccines with effective protection against homologous and some heterologous PRRS strains [14]. The criteria used to evaluate the efficacy of MLV vaccines based on clinical (clinical symptoms), virological (amount of viral load in the blood), and immunological parameters (Interferon (IFN)- γ response). Previous studies have shown that MLVs were able to prevent virus replication in PRRS target cells, viremia, and clinical symptoms by inducing antibodies to neutralize the virus [19,20]. In particular, their use in PRRSV vaccination protocols has led to a significant reduction in abortions, estrus returns, the number of stillborn and mummified piglets, and significantly increased farrowing rates and the number of live-born piglets. In addition, induction of the IFN- γ response by MLV vaccines may result in a reduction of viremia, as IFN- γ is known to inhibit PRRS replication in macrophages [20,21,22]. Thus, MLV vaccines are the dominant vaccines in the field today.

Many review articles have already been published on the protective immune mechanisms of MLV vaccines but there has been less discussion about their overall efficacy against various PRRS strains in the later stages of gestation. Specifically, many concerns have been raised as the vaccine virus has been replicated in inoculated animals and, subsequently, transmitted to naïve animals [20,23]. Moreover, many researchers have demonstrated the ability of the virus to cross the placenta during late pregnancy and cause mummification and stillbirth. However, the swine epitheliochorial placenta is considered as a barrier to the passage of antibodies from the sow to the fetus. Given that IgG antibodies (12 nm) are smaller than PRRS virion (55 nm), the chance of transferring the free PRRSV particle to embryos is very low [9,24]. Nonetheless, due to the presence of PRRS target cells in the endometrium and placenta (Sialoadhesin (Sn)+ macrophages and CD163+ macrophages), the virus crosses the uterine epithelium and trophoblast and reaches the placenta of the fetus [25,26,27,28,29]. Piglets born from these infected sows may be carriers of PRRS and then transmit the virus to other naïve pigs [30,31,32].

Despite their undesirable effects, MLVs are considered to be the key component in controlling PRRS. Therefore, it is crucial for pig producers to implement an appropriate vaccination scheme that will reduce the severity and frequency of virus-related problems. Vaccination protocols usually comprise the initial immunization of gilts and the subsequent inoculation of sows. Gilts are initially vaccinated twice (e.g., at 180th + 210th days of their lives) before being introduced into the breeding herd in order to develop complete immunity to PRRSV. As for sows, they are either inoculated with a general (“blanket”) vaccination strategy (3–4 times/year) or, depending on the breeding status, on the 6th day after birth and on the 60th day of pregnancy. Both vaccination schemes are capable of eliciting adequate immunization on the farm. Nowadays, the majority of pig farms apply the ‘mass’ or ‘blanket’ vaccination strategy in breeding female animals. However, there are some critical points in this vaccination protocol and non-compliance with them could lead to PRRSV infection. Primarily, the application of blanket vaccination involves the risk of inoculating sows in the last stage of gestation and, consequently, a plausible infection of the embryos [4,14,20,23]. Under field conditions, in our experience, PRRS MLV vaccinations are carried out even after the 100th day of gestation on many farms.

Swine farms applying PRRS MLV vaccinations without considering the critical points described above are at risk of developing fetal or neonatal pig infection due to the replication of vaccine virus. However, there is limited literature available on the side effects of PRRS MLV blanket vaccination of sows in the late stage of gestation on the health of their offspring [9,20,22,33,34]. Therefore, the aim of this study was to evaluate, whether the vaccine virus in these PRRS MLV vaccinated swine herds can congenitally infect fetuses and affect their health and immunity status

## 2. Materials and Methods

### 2.1. Ethical Approval

All procedures were done according to the ethical standards in the Helsinki Declaration of 1975, as revised in 2000, as well as the national law and after receiving approval (nr: 65/26 February 2019) from our Institutional Animal Use Ethics Committee.

### 2.2. Description of the Farms

The present study was conducted in four different commercial farrow-to-finish pig farms. The capacity of farms was about 400–600 sows under production (commercial hybrids of Large White x Landrace). All farms included similar facilities and had their own grandparent nucleus of sows to produce their own replacement gilts. Throughout the study, they did not purchase gilts from external sources. All study farms had their own feed mill and artificial insemination laboratory. The feed provided to the animals was self- prepared based on a corn/barley/wheat–soy meal, depending on the season. They also implemented regular feed mycotoxin screening schemes throughout the course of the study. Drinking water was provided for ad libitum consumption by the animals. The housing facilities had a fully automated temperature and humidity control system. In addition, all four farm-owners agreed to participate in the study voluntarily.

### 2.3. Farms’ History

All farms in the current field trial had a history of severe PRRSV epidemics over the past decade. PRRSV circulation was confirmed by real-time polymerase chain reaction (RT-PCR) testing in serum samples of all ages (sows/gilts, weaners, growers, and finishers). Clinical signs of previous PRRSV outbreaks on these farms included reproductive failure (increased abortions, increased number of mummified or stillborn piglets, and increased estrus returns) as well as severe respiratory symptoms in the nursery associated with serious economic losses. Since the first PRRSV outbreak, farms applied vaccination against PRRS for more than a decade. Experimental farms implemented a different routine vaccination program against PRRS, using different commercial MLV vaccines. In particular, gilts of all farms were initially vaccinated twice (e.g., at 180th + 210th days of their lives) before being introduced into the breeding herd. Sows of farms 1 and 3 were inoculated depending on the breeding status, on the 6th day after birth and on the 60th day of pregnancy. In contrast, sows of farms 2 and 4 were inoculated with a general (“blanket”) vaccination strategy (3–4 times/year). Farm 1 administered Vaccine One (VAC1)-Porcillis PRRS (Merck Sharp and Dohme (MSD) Animal Health, strain DV) on a routine basis, farm 2 administered Vaccine Four (VAC4)-Suvaxyn PRRS (Zoetis, strain 96V198) on a routine basis, farm 3 administered Vaccine Three (VAC3)-ReproCyc PRRS (Boehringer Ingelheim Vetmedica, strain 94881) on a routine basis, and farm 4 administered Vaccine Two (VAC2)-UniStrain PRRS (Hipra, strain VP- 046 BIS) on a routine basis. In addition, the implemented vaccination schemes on the study farms included only MLV vaccination of gilts and sows, but not of weaned ones. During the field trial period, blood samples were collected from four age groups: breeding stock (gilts, sows during early gestation and lactation), weaners at 50–60 days of age, growers at 100–110 days of age, and fatteners at 150–160 days of age. Blood samples obtained from all farms were all RT-PCR negative for PRRSV. Consequently, based on the aforementioned results, the applied vaccination protocol appeared to successfully control the circulation of the virus.

### 2.4. Experimental Design/Sample Collection/Records

The study included 25 gilts RT-PCR negative for PRRSV (five gilts per group in each farm) from each farm (25 × 4 = 100 gilts), which were equally allocated to five different study groups (five gilts in each group), as shown in Table 1. As PRRSV can cross the placenta after the 95th day of gestation [30,31,32,35], we designed an additional vaccination on the 100th day of pregnancy with different commercial PRRS MLV vaccines available in the Greek market (Table 1).

**Figure 1:**
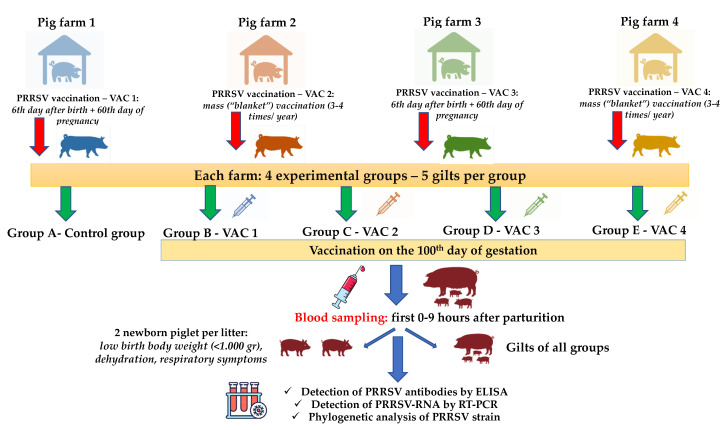
An overview of experimental design, samplings, and tests performed. VAC1: Vaccine One, VAC2: Vaccine Two, VAC3: Vaccine Three, VAC4: Vaccine Four, PRRSV: Porcine Respiratory and Reproductive Syndrome Virus, and RT-PCR: Reverse Transcription- Polymerase Chain Reaction.

The study groups were housed in different barns with separate air spaces. The animals of all groups were kept under similar conditions in terms of climate, ventilation, temperature, and air humidity. Blood samples were collected during the field trial. They were obtained from gilts by puncture of the external jugular vein using vacutainer tubes and 14 gauge (G) needles, within the first 2 h after their parturition (25 study gilts on each farm in a total of 100 blood samples) (Figure 1). Blood samples were also taken from two of the piglets of each trial gilt litter that showed poor clinical performance by puncture of the external jugular vein using vacutainer tubes and 19G needles, in the first hours after birth (50 study piglets on each farm in a total of 200 serum samples). Each newborn piglet was ear-tagged and the time between their birth and blood sampling was recorded. The criteria used to characterize the clinical performance of a newborn piglet as “poor” included low birth body weight (less than 1.000 g), dehydration, and the presence of respiratory symptoms (Figure 1). All blood samples were centrifuged for 10 min at 3000 revolutions per minute (rpm). The serum was taken and stored at −80 °C for further laboratory analysis. Moreover, blood samples were collected from the following age groups of each farm in order to investigate the circulation of wild-type PRRSV in the herd: breeding stock (gilts, sows during early gestation and lactation), weaners at 50–60 days of age, growers at 100–110 days of age, and fatteners at 150–160 days of age.

An individual examination of clinical signs in study gilts was performed daily, starting from the day before vaccination (99th day of their gestation) until the day of their parturition. The clinical observation score included assessment of behavior, respiratory signs, and fever. Local injection site reactions were investigated on the day of vaccination 0 h, 1 h, and 4 h after vaccination, and then daily until their parturition. The injection sites were examined for redness, swelling, heat, and pain on palpation. In addition, reproduction performance parameters were recorded from all gilts. Specifically, litter characteristics (healthy live born piglets, weak-born piglets, and mummified and crushed piglets per litter), abortion rate, and percentage of sows returning to estrus were noted. At birth, the piglets of the study were clinically examined daily, starting from the day they were born until the day of their weaning. The clinical observation score included assessment of behavior, digestion disorders, and, especially, respiratory signs. Piglets with poor performance, dehydration, diarrhea, neurological signs, splay-leg, and respiratory symptoms were recorded.

### 2.5. Laboratory Examinations

#### 2.5.1. Detection of PRRSV-RNA by RT-PCR and ORF5 Sequencing

Viral RNA was purified from all serum samples by using RNeasy Mini kit (Qiagen, Hilden, Germany) in automatic robot (QIAcube, Qiagen) following manufacturer’s instructions. RNAs were examined by SYBR Green real-time (ReTi) reverse transcription polymerase chain reaction (RT-PCR) for PRRSV, according to Martinez et al. [36]. Three different primer-pair RT-PCR protocols (2 European and 1 North American previously described [37,38,39] were adapted to SYBR Green methodology and used to amplify complete Open Reading Frame 5 (ORF5) gene of all the samples. The PCR products were purified and sequenced using adapted Sanger methodology [40] and nucleotide sequences were analyzed using Geneious Pro software (Biomat-ters, Ltd., Auckland, New Zealand), resulting in a 606 nucleotides alignment for ORF5 gene. The entire procedure was performed in Diagnos-HQ (Laboratorios Hipra, Amer, Girona, Spain).

#### 2.5.2. Detection of PRRSV Antibodies by ELISA

Serum samples were also tested for PRRSV-specific antibodies. Indirect enzyme-linked immunoassay (ELISA) assays were performed using the commercial CIVTEST SUIS PRRS E/S^®^ PLUS and CIVTEST SUIS A/S^®^ kits (Laboratorios Hipra, Amer, Girona, Spain). These kits were designed to detect circulating antibodies against PRRSV-1 and PRRSV-2, respectively. The test procedures and the interpretation of the results followed the manufacturer’s instructions. Samples with a relative index (expressed as a percentage) greater than 20 were considered positive [41].

### 2.6. Statistical Analysis

Data associated with the reproductive characteristics of 100 sows after parturition were available. Antibody (Ab) levels from a total of 200 sera samples from piglets from four different farms were also available. The measured variables of gilts reproductive performance and piglets’ clinical performance (and their categories) are: early birth, days of pregnancy, number of total born/alive born/dead/mummified and weightless piglets, and number of piglets with splay-leg/respiratory symptoms.

The objective of the analysis was to apply a linear regression model that predicts the antibody level response, given covariate information, which are statistically significant variables [42]. The explanatory variables inspected as potential predictors for the Ab level in this study were the following: (i) PCR sample binary result; and (ii) time interval between piglet birth and sample collection, as a categorical variable (0–3 h: 0, 3–6 h: 1, >6 h: 2). The four sampled farms apply different commercial vaccines against PRRSV.

All candidate variables were initially screened, one by one, with a significance level of 0.25. Variables with *p* < 0.25 were then offered to the full model which was, subsequently, reduced by backwards elimination, until only significant (*p* < 0.05) variables remained. The slope coefficient is the reported estimate and 95% confidence intervals (CIs) for each coefficient were constructed. The coefficient represents how the response variable changes for any change in this predictor, given that all the other predictors included in the model are held constant.

Most potential risk factors are categorical explanatory variables with more than two levels. Given the 0.05 significance level set at the full model (0.25 at the initial screening), if one level of the risk factor has a P-value above the significance level, while the *p*-values for the rest of the levels are less than the significance level, the predictor is considered statistically significant.

## 3. Results

The objective of the present study was to investigate the effects of the application of the four commercial PRRS-1 MLV vaccines in pregnant gilts (at late stage of gestation) and its potential correlation with the health and immune status of their piglets. Therefore, we conducted an evaluation of the undesirable effects of late PRRSV MLV vaccination of pregnant gilts. The criteria used to assess the potential adverse effects included the possible congenital virus infection and viremia in gilts and their litters, the reproductive performance of gilts and the health status of their offspring. Furthermore, during the pre-trial period according to RT-PCR tests performed on serum samples of different age groups (breeding stock, weaners, growers, and fatteners) on experimental farms, no positive results were detected indicating that there was no wild-type PRRSV circulation in the herds.

### 3.1. Clinical Evaluation and Reproductive Performance

Clinical observations showed the absence of local or systemic reactions as well as adverse effects on gestation after vaccination in all gilts. No abortions were recorded in all groups, while two premature farrowings were noticed in one gilt from farm 1 (group C-farrowing at 112th day of gestation day) and in one gilt from farm 2 (group D-farrowing at 113th day of gestation day). Descriptive statistics for each measured variable of reproductive performance of gilts from all trial farms and study groups are displayed in Table 2.

At farm 1, piglets born in study groups A, B, D, and E showed good average viability. Conversely, piglets born in study group C showed poor clinical performance associated with an increased average number of weak born piglets (WBPs), mummies and piglets with splay-leg (SLP) and/or respiratory signs (RSP). For farm 2, piglets born in study groups A, B, C, and E showed better clinical performance than those born in group D which was considered poor based on the number of WBPs, mummies, and SLP and RSP piglets. Finally, all piglets born in study groups from farms 3 and 4 showed good average viability clinical performance without significant differences with control groups (3A, 4A) in the number of WBPs, mummies, and SLP and RSP. Descriptive statistics for each measured clinical performance variable of the study piglets from all trial farms are presented in Table 3.

### 3.2. Laboratory Results

#### 3.2.1. Detection of PRRSV-RNA in Gilts and Piglets

**Figure 2:**
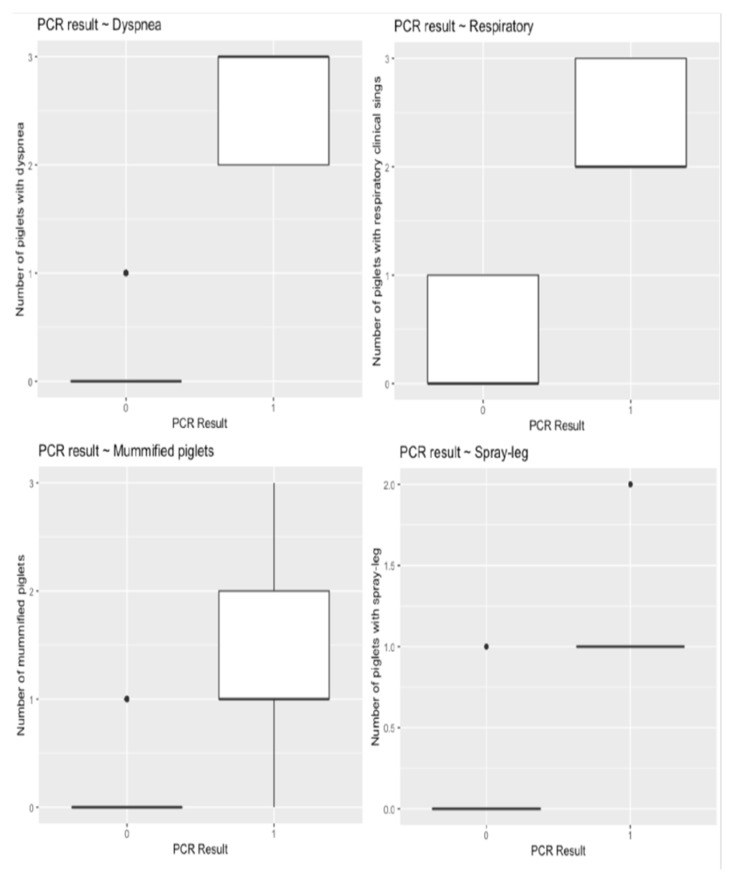
Boxplots displaying the distribution of selected outcome variables (a. dyspnea, b. respiratory, c. splay-leg, and d. mummified) given the PCR result (PCR = 0 → Negative, PCR = 1 → Positive).

Serum samples from all study gilts were tested negative for PRRSV by SYBR Green ReTi RT-PCR. A small percentage of the samples (10% = 20/200) were positive by RT-PCR. RT-PCR positive PRRSV serum samples obtained from piglets born in group C gilts, on farm 1. The remaining RT-PCR positive serum samples were collected from piglets born in group D gilts, on farm 2. Figure 2 displays the difference between the number of piglets with dyspnea, respiratory signs, splay-leg, and the number of mummified piglets, given the result of the PCR examination. Dyspnea, respiratory signs, splay-leg, and fetal mummification are more observed in piglets with positive PCR result.

#### 3.2.2. Phylogenetic Analysis of PRRSV Strain

Phylogenetic analysis was performed on positively tested samples. The analysis revealed that, on farm 1, the PRRSV field strain (GR 2019-1) showed 98.7% similarity to the PRRS MLV vaccine strain VP- 046 BIS (VAC2). In addition, on farm 2, it was revealed that the PRRSV field strain (GR 2019-2) showed 99.2% similarity to the PRRS MLV vaccine strain 94881(VAC3).

#### 3.2.3. Detection of PRRSV Antibodies in Gilts and Piglets

**Figure 3:**
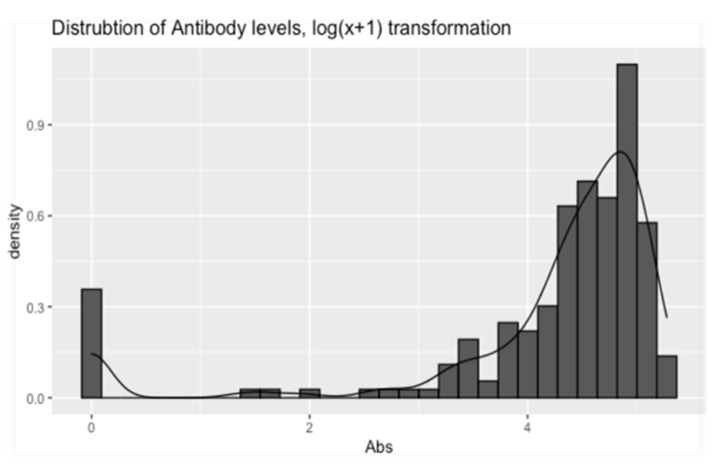
Shows the transformed (log(x + 1)) distribution of the piglets’ Ab levels. The figure was constructed using the “ggplot” function from the “ggplot2” R-package [43], in the R programming language. Abs: Antibodies.

**Figure 4:**
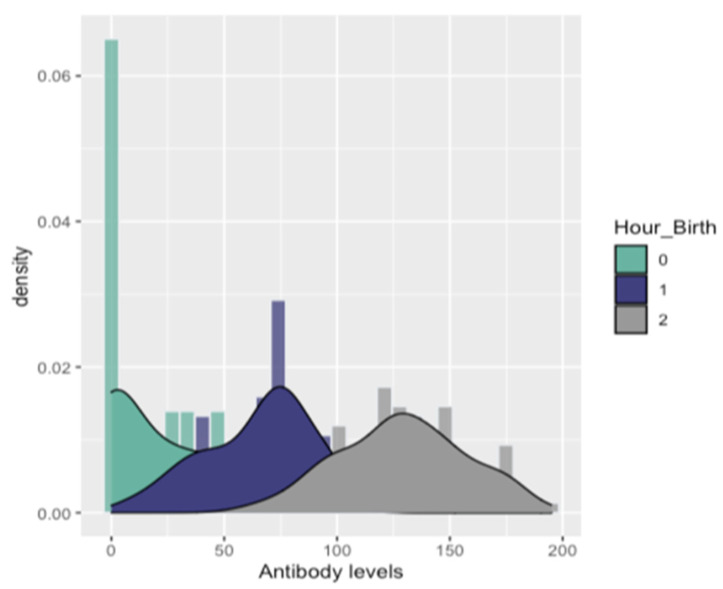
Fitted densities to the distribution of Antibodies for each level of Hour_birth [0: 0–3 h, 1: 3–6 h, and 2: > 6 h].

All ELISA tests were positive on serum samples taken from trial gilts. In addition, the majority of ELISA tests were positive for serum samples taken from each study piglet. Specifically, 181 of the total 200 serum samples were found positive (181/200 = 90, 5%) (Figure 3). On farm one, nine out of 50 serum samples (five study piglets from group C, two piglets from group D, and two piglets from group E), on farm two, four out of 50 sera samples (two study piglets from group C and two piglets from group D) and on farm four, six of 50 serum samples (four study piglets from group A and two piglets from group D) were found negative. PRRSV-specific antibodies were detected in both RT-PCR negative and RT-PCR positive piglets (Figure 3). Antibody levels were also detected when serum samples were collected immediately after birth of experimental piglets (0–3 h), when obtained a few hours after birth (3–6 h) and when taken several hours after birth (6–9 h). Descriptive statistics for antibody levels detected in study piglets are displayed in Table 2, Table 3 and Table 4 and Figure 4.

## 4. Discussion

PRRSV vaccination has been established as an important tool for minimizing reproductive failure in breeding animals and respiratory diseases in growing pigs. Today, PRRS MLV vaccines are considered to be the dominant ones in the field, as they are more effective in reducing the duration of viremia, virus shedding, disease occurrence, and severity compared to the PRRS KV vaccines [14,15,17,19,20,21]. Despite their significant contribution to virus control, many concerns have been reported about the outcomes of MLV vaccination, especially when applied to a blanket vaccination strategy. However, only a limited number of studies have been conducted to identify the potential risks of the PRRS MLV mass vaccination strategy [9,14,23,33,34]. In our study, we used a pregnant sow model and exposed gilts to the PRRS-vaccine virus at 100 days of their gestation, using all four different commercially available vaccines containing attenuated PRRSV-1. The criteria used to evaluate the potential adverse effects of MLV vaccines on this model were based on virus congenital infection and viremia in gilts and their litters, the reproductive performance of gilts, and the health status of their offspring.

Primarily, the study showed that the exposure of the experimental gilts to the vaccine strains appeared to be safe. The absence of local or systemic clinical signs, such as fever, anorexia, and reproductive failure, were observed in vaccinated sows during the late stage of gestation. Specifically, abortions were not recorded and a low percentage of study gilts (10/100 = 10%) farrowed early. In addition, all study gilts were found RT-PCR negative. Failure to confirm viremia in tested animals may be related to its short duration that could be missed due to our sampling schedule, or to a low level of viremia that could not be detected. This hypothesis is consistent with previous studies describing similar cases where blood samples were obtained nine days after the MLV vaccination of gilts and due to short-term viremia, the virus could not be detected [4,20]. According to Peltoniemi et al. [44], blood sampling is a stress factor for gilts that could even lead to reproductive disorders (e.g., abortions, and estrus returns). Thus, blood sampling of our trial gilts was conducted shortly after their parturition to avoid the potential occurrence of such problems (15 days after PRRS MLV vaccination). Furthermore, PRRSV antibodies were detected in all study gilts after PRRS MLV vaccination. These results can be explained by the fact that vaccination process can lead to high levels of antibodies in animals that have been previously inoculated [15,45]. Several studies have also reported the presence of PRRS antibodies in pregnant gilts after MLV vaccination [20,22,46]. In particular, Mada pong et al. [47] performed ELISA tests after PRRS MLV inoculation of pregnant gilts and observed the presence of antibody responses 7–14 days post-vaccination (DPV) and persisted at high levels until 84 DPV.

Despite the lack of viremia in the study gilts, we observed congenital infection and viremia in 20 piglets. Specifically, farm 1 piglets born in study group C exposed to infectious strain VP- 046BIS (VAC2) and farm 2 piglets born in study group D exposed to infectious strain 94881 (VAC3) were RT-PCR positive. Subsequently, we identified, using the ORF 5 sequence, that the PRRSV strains detected in these piglets showed a high degree of similarity to the vaccine strains in which the farm 1 group C gilts (PRRSV field strain 98, 7% similarity with MLV vaccine strain of VAC2) and the group D gilts from farm 2 (PRRSV field strain 99, 2% similarity with MLV vaccine strain of VAC3) were exposed to. Our results corroborated previous findings by Karniychuk and Nauwynck [18], who identified how PRRSV spreads from mother to embryos and the pathophysiological basis of viral-induced reproductive failure. Their survey indicated the involvement of the endometrium and placenta in the transmission of the virus to fetuses and the main role of macrophages Sn+ CN163+ in the reproduction of the virus. They concluded that the replication of the virus in the endometrium and placenta in late gestation is responsible for the possible congenital infection. In addition, many researchers have reported the birth of viremic piglets after PRRS MLV vaccination of gilts in the last trimester of gestation, claiming that PRRS MLV inoculation led to a congenital infection and the virus was transmitted to fetuses [9,22,33,34,47,48]. However, through phylogenetic analysis of ORF5 in our study, we demonstrated the ability of VAC2 and VAC3 strains to cross the placenta and infect fetuses. Consequently, the PRRS MLV vaccination of gilts on the 100th day of gestation resulted in a congenital infection and the birth of viremic trial piglets.

Moreover, a previous study reported that PRRSV MLV vaccination of pregnant gilts is likely to lead to increased transfer of maternal antibodies in piglets and reduction in the proportion of viral neonates [49]. Thus, given the difference in the distribution of PRRSV-positive piglets over blood sampling time, the findings of the present study are consistent with the claims of this previous study [49]. In particular, 37.5% (12/32 = 37, 5%) of piglet blood samples taken immediately after birth (0-3 h), without receiving any proportion of colostrum, were PRRSV positive. In contrast, only 4.7% (8/168 = 4, 7%) of the remaining trial piglets which had suckled and consumed colostrum, were found to be PRRSV-positive. Finally, no PRRSV- infected study piglets were detected on farms 3 and 4. This may be associated with many factors such as genetic susceptibility, environmental conditions, housing system, and mode of production (e.g., female introduction practices) [46]. In addition, recent studies have reported that PRRS vaccine strains show similar genetic diversity and instability to their natural “wild” strains, resulting in a high rate of mutation [50]. Therefore, it is tempting to suggest that several strains of PRRS MLV vaccines may have become more infectious, inducing congenital infection and viremia in piglets.

Regarding the ELISA assays performed on the newborn piglets of the study, PRRSV antibodies were detected in both RT-PCR positive and negative piglets. In addition, statistical analysis showed significantly higher mean antibody levels in piglets whose serum samples were collected 6–9 h after birth, compared with piglets whose serum samples were collected immediately (0–3 h) or shortly after birth (3–6 h). Most of the piglets were blood sampled up to 3 h after birth did not have enough time to suckle and did not receive colostrum, unlike to rest trial piglets that ingested high concentrations of colostrum. Our results partially support previous studies, which have shown that neonatal piglets receive passive immunity only after birth, by ingestion of colostrum, as the porcine placenta inhibits the in utero antibody transfer [51,52,53,54,55]. In particular, the epitheliochorial placenta of swine is considered to be a barrier to the transfer of maternal antibodies to embryos. The porcine placenta presents the greatest degree of separation between the maternal and the embryonic circulation, compared to other mammals, as it consists of six different layers: maternal blood vessels, endometrial connective tissue, uterine epithelium, trophoblast, fetal placenta mesenchyme, and fetal blood vessels. As a result of this firm barrier between maternal and fetal blood, antibodies cannot pass to the fetuses during gestation [18,52,53,56,57]. Consequently, the amount of maternal immunity in neonatal piglets depends on the concentration of colostrum ingested. Thus, the differences revealed in the antibody levels between our study piglets can be explained by the different proportion of colostrum they consumed. Nevertheless, in contrast to previous researchers who reported that no antibodies are transferred through the placenta from the dam to the fetus before birth, we found PRRSV antibody levels in non-suckling piglets. Butler and colleagues [58] indicated that maternally derived antibodies could enter the fetal circulation because of de novo synthesis. They suggested their presence because of environmental antigens such as the virus, which can cross the porcine placenta, just like PRRSV, and their subsequent de novo synthesis. These findings in association with PRRS viremia detected in several piglets sampled before lactation (12/32 = 37, 5% of these piglets were RT-PCR positive) support our results regarding the presence of PRRSV specific antibodies in study piglets. In contrast, the presence of PRRSV antibodies in the other study piglets may be justified by the fact that at the time of sampling (3–6/6–9 h after birth) they had managed to suckle and receive colostrum.

There are published evidence that MLV vaccines elicit specific humoral and cell-mediated immune (CMI) responses, as they can offer efficient protection to homologous strains and partial protection to heterologous strains [59]. Innate immune responses against PRRSV are obstructed by different mechanisms as are adaptive responses. The modest and delayed B cell mediated neutralizing antibody response is one of the main characteristics associated to PRRSV acquired immune responses. Even though PRRSV induces an antibody response (production) by 7–9 days post infection (DPI), the efficacy of those antibodies remains unclear; serum neutralizing antibodies appear only later, typically ≥28 DPI [60]. The virus also evades host cell-mediated immunity most likely by the promotion of immunosuppressive cytokines (Interleukin (IL)-10 and Transforming Growth Factor (TGF)-β resulting in delayed onset of Th1 immune response [61]. Studies reported that passive transfer of high neutralizing antibody titter conferred protection to gilts and offspring (not detectable viremia) but did not eliminate the presence of viral particles in peripheral tissues (82–84). However, the role of neutralizing antibodies in the protection against the respiratory form of the PRRS disease is a key gap of knowledge [59,60,61]. In contrast to the previous studies, the presence of PRRSV specific antibodies was detected in several RT-PCR positive (viremic) piglets that did not receive colostrum in our trial. However, no evaluation of CMI was performed in our study. Further studies should be of great interest for future trials, including PRRS MLV vaccination in the late stages of gestation.

As for the clinical data of the study piglets, we observed a significantly lower average number of live-born piglets per litter in groups 1C, 1D, and 2D compared to the other study groups. In addition, in each of these groups there were also slightly higher mean numbers of DBP and/or WBP, and several SLP and/or RSP, or even mummified piglets–more than in the other groups and certainly more than in the four control groups (A) without late PRRSV vaccination (Table 2, Figure 2). The birth of mummified and low-bodyweight piglets, as well as piglets with splay-leg or respiratory problems, are typical clinical signs caused by the epizootic form of PRRSV on farms [7,62,63,64]. Several research ers have also described clinical cases of PRRSV-infected piglets, which showed a variety of clinical symptoms, such as lethargy, apathy, low bodyweight (BW < 1000 gr), splay-leg, and respiratory problems [62,63,64,65,66]. Therefore, our findings can be justified by the reports of the above studies in combination with the viremia detected in several trial piglets. However, the data obtained from our study contradict the claims of the researchers who demonstrated that despite placental infection of fetuses due to late PRRS MLV vaccination, the infectious strains had little or no direct detrimental effect on the piglets [4,24,50]. This disagreement may be related either to the virulence of infectious strains or to other factors such as different genetic susceptibility, housing system, environment, or the production type (e.g., female introduction system) of farms [1,49]. Additionally, in our study the PRRS MLV vaccination was administered on the 100th day of gestation, while in previous trials it was applied on the 90th day of pregnancy. Thus, different PRRSV vaccination times may also be associated with this contradiction between study results.

## 5. Conclusions

Our study demonstrates that PRRSV vaccine strains can act as an infectious strain when PRRS MLV vaccination is applied in late gestation, affecting the health and immune status of piglets. We revealed the occurrence of PRRSV congenital infection and viremia in piglets during their uterine life in PRRS MLV vaccinated gilts. Furthermore, the passage of specific PRRSV antibodies into the circulation of congenital infected piglets could be attributed to *de novo* synthesis. Finally, piglets congenitally infected with PRRS MLV strains showed poor clinical performance compared to the rest of the non-PRRS MLV infected piglets. Therefore, our study provides strong evidence that suggests serious risks that may arise from late PRRS MLV vaccination of gilts. According to our findings, pig farms applying PRRS MLV vaccination to a routine blanket vaccination strategy should avoid vaccinating pregnant gilts in the last two weeks before parturition. Although PRRSV vaccination does not necessarily provide complete protection against virus infection, it is the pillar on which a PRRSV control plan should be based. Thus, it is crucial to adhere to the critical points in the various PRRSV vaccination protocols in order to avoid potential risks from their implementation. For the future, large-scaled studies are required to further investigate the potential ability of PRRS MLV vaccines to congenitally infect fetuses, induce viremia and cause health disorders when being administered before the 100th day of gestation.

## Figures and Tables

**Table 1:** Experimental groups and experimental design.

Experimental Farms	Group	Vaccination on the 100th Day of Gestation
**Farm 1** (Gilts were vaccinated with VAC1 180th + 210th day of age + 60th day of gestation)	A	None–Control group
	B	Vaccine One (VAC1)-strain DV
	C	Vaccine Two (VAC2)-strain VP- 046 BIS
	D	Vaccine Three (VAC3)-strain 94881
	E	Vaccine Four (VAC4)-strain 96V198
**Farm 2** (Gilts were vaccinated with strain VAC4 180th + 210th day of age + 60th day of gestation)	A	None–Control group
	B	Vaccine One (VAC1)-strain DV
	C	Vaccine Two (VAC2)-strain VP-046 BIS
	D	Vaccine Three (VAC3)-strain 94881
	E	Vaccine Four (VAC4)-strain 96V198
**Farm 3** (Gilts were vaccinated with VAC3 180th + 210th day of age + 60th day of gestation)	A	None–Control group
	B	Vaccine One (VAC1)-strain DV
	C	Vaccine Two (VAC2)-strain VP-046 BIS
	D	Vaccine Three (VAC3)-strain 94881
	E	Vaccine Four (VAC4)-strain 96V198
**Farm 4** (Gilts were vaccinated with strain VAC2 180th + 210th day of age + 60th day of gestation)	A	Non–Control group
	B	Vaccine One (VAC1)-strain DV
	C	Vaccine Two (VAC2)-strain VP-046 BIS
	D	Vaccine Three (VAC3)-strain 94881
	E	Vaccine Four (VAC4)-strain 96V198

**Table 2:** Antibody level statistics and RT-PCR results for each category/level of Hour_birth.

Antibody Levels		1st Quantile (25%)	Mean	Median	3rd Quantile (75%)	RT-PCR Status	
						Positive	Negative
Hour_birth	0–3 h	0	14	23.7	45.5	12	20
	3–6 h	45.5	64.6	70.5	80.5	8	48
	>6 h	108	129	128	148	0	112

RT-PCR: Real Time- Polymerase Chain Reaction.

**Table 3:** Descriptive statistics (mean/median (min–max)) for all measured variables for each farm and group combination.

Experimental Farms	Group	DT (Days)	Number of Piglets Mean/Median (Min–Max)					
			LBP	DBP	WBP	Mummies	SLP	RSP
**Farm 1**	**A**	118.4/119 (117–119)	16/16 (14–18)	1.4/1 (1–2)	0.8/1 (0–1)	0/0 (0–0)	0/0 (0–0)	0/0 (0–0)
	**B**	117.4/117 (116–119)	13.4/13 (11–17)	1.8/2 (1–3)	1.6/1 (1–3)	0.4/0 (0–1)	0/0 (0–0)	0.8/1 (0–1)
	**C**	114.2/115 (112–116)	9.8/10 (7–12)	2.2/2 (2–3)	3/3 (2–4)	2/2 (1–3)	1.2/1 (1–2)	2.4/2 (2–3)
	**D**	116.8/117 (116–118)	8.8/10 (7–10)	1.6/2 (1–2)	1.6/2 (1–2)	0.4/0 (0–1)	0.4/0 (0–1)	1/1 (1–1)
	**E**	119.2/119 (118–120)	15/15 (14–16)	1.8/2 (1–3)	2/2 (0–4)	0/0 (0–0)	0/0 (0–0)	0.6/1 (0–1)
**Farm 2**	**A**	117/117 (116–118)	16.4/16 (16–17)	1.8/2 (1–2)	1.4/1 (0–3)	0/0 (0–0)	0/0 (0–0)	1/1 (1–1)
	**B**	117/117 (116–118)	8.2/8 (5–11)	3.8/4 (3–5)	1.4/1 (1–2)	0/0 (0–0)	0/0 (0–0)	1/1 (1–1)
	**C**	117/117 (116–118)	12.4/12 (10–15)	4.4/4 (2–7)	2.4/2 (2–3)	0.6/1 (0–1)	0.4/0 (0–1)	1/1 (1–1)
	**D**	114.8/115 (113–117)	5.6/6 (4–7)	1.4/1 (1–2)	2.4/2 (2–3)	0.6/1 (0–1)	1.2/1 (1–2)	2.4/2 (2–3)
	**E**	116.2/116 (115–118)	14.6/14 (12–18)	1.2/2 (0–2)	2.4/2 (2–3)	0/0 (0–0)	0/0 (0–0)	0/0 (0–0)
**Farm 3**	**A**	116.6/117 (116–117)	12.8/13 (12–14)	1.6/2 (1–2)	0/0 (0–0)	0/0 (0–0)	0/0 (0–0)	0/0 (0–0)
	**B**	116.4/116 (116–117)	13.4/13 (12–15)	1/1 (0–2)	0/0 (0–0)	0/0 (0–0)	0/0 (0–0)	0/0 (0–0)
	**C**	116.6/116 (115–119)	13.4/13 (11–16)	0.6/1 (0–1)	0/0 (0–0)	0/0 (0–0)	0/0 (0–0)	0/0 (0–0)
	**D**	117/117 (116–118)	12.2/12 (11–13)	1.2/1 (1–2)	0/0 (0–0)	0.6/1 (0–1)	0/0 (0–0)	0/0 (0–0)
	**E**	117/117 (116–118)	14.4/14 (14–15)	1/1 (0–2)	0/0 (0–0)	0/0 (0–0)	0/0 (0–0)	0/0 (0–0)
**Farm 4**	**A**	117.4/117 (117–118)	13/13 (11–15)	1.6/2 (1–2)	0.6/1 (0–1)	0/0 (0–0)	0/0 (0–0)	0/0 (0–0)
	**B**	117.4/117 (117–118)	11.6/12 (10–13)	1.4/1 (1–2)	0.6/1 (0–1)	0/0 (0–0)	0/0 (0–0)	0.4/0 (0–1)
	**C**	116.4/116 (116–117)	11/11 (10–13)	1.6/2 (1–2)	0.6/1 (0–1)	0/0 (0–0)	0/0 (0–0)	0.6/1 (0–1)
	**D**	117.4/117 (117–118)	11.8/12 (10–14)	2.6/3 (2–3)	1.4/1 (1–2)	0/0 (0–0)	0/0 (0–0)	0.6/1 (0–1)
	**E**	117/117 (116–118)	14/14 (13–16)	2.4/2 (2–3)	1/1 (0–2)	0/0 (0–0)	0/0 (0–0)	0.6/1 (0–1)

DT: Duration of gestation, LBP: Live Born Piglets, DBP: Dead Born Piglets, WBP: Weak Born Piglets, SLP: piglets with splay-leg, and RSP: piglets with respiratory signs.

**Table 4:** Estimates and 95% confidence intervals (CIs) for the effect of covariates on Antibody levels.

Linear Regression			
Parameter	Hour after birth	Estimate (95% CI)	*p* Value
**Hour** **Birth**	0–3 h	4.3 (2.75; 6.71)	<0.001
	3–6 h	8.26 (5.77; 11.8)	<0.001
	6–9 h	26.33 (17.23; 40.24)	<0.001

## Data Availability

Not applicable.
